# Pituitary Disorders and COVID-19, Reimagining Care: The Pandemic A Year and Counting

**DOI:** 10.3389/fendo.2021.656025

**Published:** 2021-03-12

**Authors:** Maria Fleseriu

**Affiliations:** Pituitary Center, and Departments of Medicine (Endocrinology, Diabetes and Clinical Nutrition), and Neurological Surgery, Oregon Health & Science University, Portland, OR, United States

**Keywords:** COVID–19, pituitary, SARS–CoV–2, medical treatment, risk

## Introduction 

It has been more than a year since the severe acute respiratory syndrome coronavirus 2 (SARS-CoV-2) “eruption” on the world scene and the coronavirus disease 2019 (COVID-19) pandemic engulfed all lives on all continents. And quite the year, was 2020!

Physicians from all specialties have been affected, both personally and professionally; from screening for and treating patients with COVID-19, which did not fall in their area of expertise, or from delaying care of patients with acute and chronic disorders with other pathologies. Moreover, COVID-19 created new pathologies and several patients with chronic conditions are at higher risk. Further complicating the picture; most non-acute care has been moved *on* and *off* from in-person clinic visits to remote telemedicine visits, while elective surgeries have been also on hold during several “pause” waves.

Pituitary diseases and several disease components, such as acute visual loss, tumor mass effects, or chronic conditions such as adrenal insufficiency (AI), hypopituitarism, Cushing’s disease (CD) or growth hormone (GH) excess (acromegaly) have had tremendous interplay with COVID-19.

Several recommendations from international experts published by the Pituitary Society (1) or the European Journal of Endocrinology, affiliated with European Society of Endocrinology (2–5) were published early in the course of the pandemic; notably, a re-evaluation every few months in light of emerging data, was suggested (1, 6). Unfortunately, at this time, despite an ongoing unprecedented vaccination campaign, the risk of viral infection load in many countries is the highest it has been and local rules for “lockdown” have been intensified rather than relaxed overall.

Furthermore, many patients without a history of pituitary disorders who have had severe SARS-CoV-2 are being treated with high dose glucocorticoids (GC). This is based on data suggesting that abnormal immune reactivity could cause additional lung damage and progression to severe respiratory failure rather than uncontrolled viral replication. Notably, dexamethasone was shown to reduce intermediate (28-day) mortality among patients on either invasive mechanical ventilation or oxygen alone in the Randomised Evaluation of COVID-19 Therapy (RECOVERY) trial (7). However, administration of anti-retroviral drugs and high dose GC use could trigger drug–drug interactions and enhance exposure to drugs that are metabolized through the CYP450/CYP3A pathway, impacting the hypothalamic-pituitary-adrenal HPA axis (8) and highlighting a need for HPA axis monitoring at discharge from the hospital.

Another interplay between COVID-19 and pituitary disorders is linked to the suggestion that many patients with fractures should start, in addition to calcium and vitamin D supplementation, early bone-targeted treatment, even while in hospital (9). This can prove complicated due to limited resources that prompt early discharge during the COVID-19 pandemic. As such, a communication plan regarding the importance of anti-osteoporosis treatment post-discharge is essential (10). Many patients with pituitary disorders who are at higher risk of fractures such as CD, acromegaly, hypopituitarism (11–14), and central AI could thus possibly need anti-resorptive treatments (15).

Here, we focus on updates on the risks associated with COVID-19 in patients with pituitary disorders. We also discuss management of patients with either new or long-standing pituitary disorders in the setting of limited healthcare delivery due to the pandemic.

## COVID-19 and Patients with Pituitary Disorders

### Cushing’s Disease (CD)

Patients with CD can experience many comorbidities, which can in turn increase complication(s) risk if patients with CD become infected with COVID-19 (1, 5). It has been recognized that the severity of COVID-19 is higher in patients with diabetes mellitus (DM) and hypertension (16) Moreover, COVID-19 might increase the risk of hyperglycemia, which can modulate immune and inflammatory responses, thus worsening the risk of severe disease (17). Complicating the picture even more, many medications used for COVID-19 treatment can affect glucose metabolism, particularly with preexisting DM; thus, both glucose monitoring and individualized management are usually needed for patients with DM and/or hypertension (16). Patients with cardiovascular or kidney disease have been also shown to have a poorer prognosis than those without these diseases and COVID-19 (18, 19). Obesity reduces respiratory system compliance, expiratory reserve volume, and functional capacity. Sleep apnea is more frequent and further impairment of pulmonary function is noted with reduced excursion of diaphragm. Several other comorbidities, including thrombosis, also have a great impact in COVID-19 infected patients’ outcomes (18). However, currently, there are no studies that answer an essential question; are these comorbidities in patients with pituitary disorders having a different impact on outcomes when compared with patients without pituitary function abnormalities (either excess or deficiency)? One could extrapolate that a high burden of cardiovascular, metabolic and respiratory comorbidities would increase overall disease severity in patients with pituitary disorders if they become infected with COVID-19.

For patients with active CD who develop COVID-19, risk of severe thromboembolism on already heightened hypercoagulability due to CS could be compounded (20), thus anticoagulation treatment *per se*, not just preventive doses should be recommended in all hospitalized patients with CS, which can decrease mortality overall (21).

Presently, there is limited data on treating patients with CS and COVID-19. Yuno et al., described a 27-year-old female with CD who was awaiting pituitary surgery when she developed COVID-19 pneumonia (22). She was treated with a “block-and-replace” regimen using a steroidogenesis inhibitors combination (high dose metyrapone, 4,000 mg/day and trilostane) and hydrocortisone, and a multimodal treatment that included antiviral therapy. The patient improved clinically and underwent curative surgery 1 month later (after testing negative for COVID-19) (22). Beretta et al., reported on a case of a 67-year-old male with CD, who presented with signs and symptoms of AI; (persistent hypotension and hypoglycemia) after he became infected with COVID-19 (23). Pasireotide and metyrapone and DM treatment were halted, and injectable GC was started. Furthermore, unexpected hyperkalemia in CS patients under treatment with heparin, as was the case in this patient, might be a signal of aldosterone suppression and increased awareness for electrolyte abnormalities is needed (23).

Titration and/or temporary halting medical therapies in the treatment of patients with CS in the context of COVID-19 infection should be considered; adjustments dependent on drug regimen and symptom severity. If patients are COVID-19 positive, but asymptomatic, an attempt should be made to keep patients eucortisolemic and avoid AI (1, 5). Patients should have at home already and available oral and injectable GC, but administration is probably not needed preemptively unless a patient develops symptoms. In our experience, we have stopped CS medication in two patients with severe COVID-19 symptoms and administered high dose GC for a few days with no long-term complications, and complete recovery in both patients *(unpublished data).*

For patients who are not on a “block-and-replace” regimen, during the height of the pandemic when emergency department and urgent care visits were limited, mild hypercortisolemia, especially for a short period of time is preferable versus AI/adrenal crisis. On the other hand, Etomidate treatment might also be needed for patients with severe newly diagnosed CD in the context of COVID-19 if hyperglycemia is uncontrolled, hypertension and infections are present, and imaging and/or definitive treatment(s) with tumor removal are postponed.

A study by Mirani et al., showed that glucose level at hospital admission and ongoing anti-diabetic drugs may influence the outcome of COVID-19 in patients with DM2 (24). Interestingly, patients on insulin therapy at admission had a more severe evolution of COVID-19 (a more than three times increased mortality risk). Patients taking Dipeptidyl peptidase-4 inhibitors (DDP4-I) had better outcomes. However, the number of patients taking DDP4-I was small. A clear conclusion cannot be determined, but this raises an interesting hypothesis, one which was also suggested in another observational study in patients with type 2 diabetes taking Sitagliptin (25). Specific data in patients with endogenous CS is lacking, but one can assume that if there is no contraindication DDP4-I should be considered in the treatment armamentarium if a COVID-19 hospitalization ensues.

Lastly, one should consider many possible interactions between the pharmacological treatment of CS and medications used to treat COVID-19 that have the potential for QTc prolongation, liver toxicity, and hypo- and hyperkaliemia (8, 23).

### Acromegaly

Patients with acromegaly have several characteristics that could potentially worsen their outcome if they develop COVID-19 (26). Patient baseline comorbidities and treatment management may also be impacted due to drug-drug interactions with some medications used to treat COVID-19. Some studies have shown a higher risk of arrhythmias in patients with acromegaly, and an increase of QTc interval is a possible side effect of octreotide, lanreotide, and pasireotide, (long-acting somatostatin receptor ligands; SRLs) treatment (26).

Prevalence of glucose abnormalities (prediabetes and diabetes) in patients with acromegaly is high, up to 50% (27), and hypertension is reported in approximately 30% of patients (27). There are no available studies in patients with acromegaly, however, data shows that fasting blood glucose (FBG) ≥ 7.0 mmol/l at admission is an independent predictor for 28-day mortality in patients with COVID-19 without a previous diagnosis of diabetes (28). This highlights the need for close monitoring and glycemic control in all COVID-19 patients.

Interestingly, a new study showed that risk is considerably decreased in patients treated with metformin prior to COVID-19 diagnosis, thus raising the idea that metformin could be somewhat protective in this high-risk population (29).

Another co-morbidity; bone disease with vertebral fractures (VFs), which could influence cardiorespiratory function and disease outcomes has a high prevalence in patients with both controlled and uncontrolled acromegaly (27, 30). A recent Italian study (114 patients admitted with COVID-19 and no known pituitary disorders) found thoracic VFs in 41 patients (36%) (31). Patients with VFs here were older and more frequently affected by hypertension and coronary artery disease, were hospitalized and more frequently required noninvasive mechanical ventilation compared with those without VFs. Furthermore, mortality was higher in patients with VFs compared to patients without VFs, and was higher in patients with severe VFs compared to those with moderate and mild VFs (31). As VF seems to be a good marker of patient fragility and poor prognosis, it will be important to continue to study patients with acromegaly who develop COVID-19 and subsequent outcomes.

### Hypopituitarism

Epidemiological studies showed a sex dimorphism (with male preponderance) and age-dependent disease susceptibility to the severity of COVID-19 disease, with young individuals experiencing overall a milder illness. The progressive functional decline and dysregulation in the immune system in both sexes with age could be the culprit (32).

#### Hypogonadism

Brandi and Giustina (32), suggest that sex-specific measures be considered in a comprehensive management plan for patients with COVID-19; adjusting testosterone replacement doses in men to avoid a pro-inflammatory response of hypogonadism and increased venous thromboembolism (VTE) typical of higher testosterone levels. For women, stopping oral contraceptives, but continuing menopausal hormone replacement therapy, preferably transdermal estrogen is recommended (32).

#### Adrenal Insufficiency

While evidence of hypothalamic-pituitary involvement by SARS in a study of 61 survivors has emerged following the 2003 outbreak (33), there is no evidence of a direct COVID-19 effect on the pituitary or hypothalamic axis. Interestingly, 40% of SARS survivors had biochemical evidence of central AI, which mostly resolved within a year (33).

Historical data on impaired immune function may suggest higher risk of complications and mortality in patients with AI. A large retrospective case-control study undertaken in Lombardy, Italy, (one of the most affected areas in Italy, in the Spring of 2020) by Carosi et al., analyzed data collected with a standardized telephone questionnaire from 279 patients with primary and secondary AI and 112 controls (with benign pituitary lesions with normal pituitary function (34). Prevalence of symptomatic patients (complaining at least 1 symptom of viral infection) was similar between the two groups (24% in AI and 22.3% in controls, p = 0.79). The exact prevalence of infections is, however, unknown as testing was undertaken in just 12 patients. However, highly suggestive COVID-19 symptoms (at least two including fever and/or cough) occurred equally in AI and controls (12.5% in both groups). No hospitalization and no adrenal crisis were reported (34). It is reassuring that patients with AI who are on adequate GC replacement and appropriately adjust stress doses do not have higher rates of COVID-19-suggestive symptoms and/or disease severity compared with controls (34).

Treatment of patients with AI under major stress to avoid adrenal crisis has been studied (35) and should be considered when treating critically ill patients with COVID-19 (2). Expert consensus has recommended oral stress-dose coverage of 20 mg hydrocortisone (HC) every 6 h to maintain a more continuous level of steroid support in a patient with known AI; given the known associated persistent inflammation and stress associated with either suspected or confirmed acute COVID-19 infection (2). The Italian Society of Endocrinology Expert opinion (36) has taken a different approach; suggesting doubling the usual steroid dose in patients with suspected COVID-19 who have mild symptoms, but increasing to 100 mg HC (parenteral preferred) if there is progresses to what is defined as moderate COVID-19, and finally a high-dose 200 mg/24 h continuous infusion if COVID-19 is defined as severe. Due to known coagulation abnormalities associated with GC use and coagulopathies observed with severe COVID-19 anticoagulation has been suggested in all of these patients (36).

#### Growth Hormone Deficiency

Several possible pathophysiologic mechanisms linking alterations in GH/IGF-1 axis and severity of COVID-19, including links to obesity have been suggested, but more research is needed (37).

#### Central Hypothyroidism

A single center retrospective study has shown that COVID-19 may be associated with a high risk of thyrotoxicosis due to systemic immune activation by the SARS-CoV-2 infection. This can potentially complicate the management of partial central hypothyroidism in some patients with COVID-19, thus close monitoring and thyroid replacement adjustment is advisable (38).

#### Diabetes Insipidus

Patients with pre-existing conditions, including endocrine disorders may be vulnerable to plasma sodium abnormalities in more severe cases of COVID-19 (3). There have been no specific published reports, just yet. Patients with DI who develop respiratory complications of COVID-19, especially those with adispic DI are at significantly increased risk of dysnatremia and desmopressin should be administered parenterally (3).

A recent expert opinion review advises how endocrinologists can optimally care for ambulatory patients with central DI and hyponatremia, when regular visits and biochemical assessments are delayed (3). There are several highlight points; 1) priority of routine treatment of central DI should be to avoid hyponatremia, thus physicians and patients should be aware of the importance of delaying desmopressin doses to allow proper free water clearance, and 2) preference of 0.9% sodium chloride use as volume restoration in patients with COVID-19 hypovolemic shock, even if there is hypernatremia. The authors suggest that in the absence of hypovolemic shock, patients with DI and severe dehydration should be treated with hypotonic fluids (3).

## Re-Envisaging Treatment and Follow-Up of Patients With Pituitary Disorders During the COVID-19 Pandemic

### Surveys of Patients With Pituitary Disorders, and Physicians Taking Care of Patients

The precise impact and measure of pandemic restrictions, even for patients who do not contract COVID-19, such as delays in diagnosis and care due to resource alteration by healthcare institutions is not known. However, a few investigators, using surveys, are attempting to determine the direct and indirect impact of the COVID-19 on patients with pituitary disease from a patient and a physician perspective.

In a large study that included 412 patients (412/586; 70.3% answered a survey) in a single center cross sectional survey in the UK (interestingly few patients actually had COVID *per se*); 66 patients (66/412; 16.0%) reported having suspected COVID-19 infection, and from 10 patients tested, just three had a positive test (39). Furthermore, no deaths due to COVID-19 were identified. Blood tests planned to assess or monitor pituitary hormone levels were not performed in almost half of patients, while few had imaging (12.4%) or delayed and planned endocrine dynamic test (1.7%). Of note is that despite only a small percentage of patients having confirmed or suspected COVID-19 infection, over half were still indirectly impacted by the pandemic through a delay or change to their planned care. Furthermore, the study included data up until summer 2020 after a “first wave” and we can assume that now in a “third wave” and with ongoing lockdowns, delay in regular care combined with number of patients affected by COVID-19 could be actually much worse. An additional worrisome trend was the spontaneously reported comments on the negative impact of the COVID-19 pandemic on their mental health (39). While the negative psychological effects of quarantine have been recognized (40) ongoing research and mitigation efforts are needed.

An international survey, focused on patients with acromegaly included endocrinologists, patients, nurses and neurosurgeons; data from endocrinologists was published (41) and interim patient data recently presented (42). Questions focused on five broad categories; 1) impact on evaluation and diagnosis, 2) access to treatment and management, 3) impact on monitoring, 4) role of technology and remote communication, and 5) future management. The majority of endocrinologists who responded (n = 84) were based in Europe (67.9%) (41). In terms of patient-perceived risk, 76.2% of endocrinologists indicated that patients had approached them asking if they had increased COVID-19 risk. Forty-one-point seven percent (41.7%) of respondents reported that their patients had actively sought advice regarding acromegaly management under pandemic conditions, and 59.5% reported that patients had sought help regarding medical therapy. Half indicated that they believed that the role of self/partner-administered SRLs plays increased role under pandemic conditions, with 33.5% of respondents recommending a switch to self/partner-administered SRLs in patients lacking biochemical control and just 9.5% of respondents recommended delaying monthly SRLs to avoid possible patient exposure to COVID-19. These results suggested the COVID-19 pandemic is substantially affecting the care of patients with acromegaly. However, 55.9% noted that remote methods improved communication with patients. The goal of these changes must be both to improve care while safeguarding patients from more severe involvement in concomitant acute illnesses such COVID-19.

Effects of the pandemic on patients living with acromegaly were similarly broad in the survey (42) with more than one-quarter of patients from 182 interviewed reporting difficulties accessing therapy; 39.0% respondents agreed or strongly agreed that the COVID-19 pandemic had affected their ability to provide samples for regular laboratory testing. Twenty-six-point nine percent (26.9%) of respondents reported limited access to injectable drug therapies and 22.0% reported access to care-team members who administer injectable therapies were affected. Just 2.8% respondents had tested positive for the SARS-CoV-2, highlighting again how much care has been affected for all patients. More than half (51.1%) agreed or strongly agreed that the pandemic had made managing their condition more challenging (42).

### Pituitary Surgery

The number of community cases and inpatients with COVID-19, and staffing shortages (43) should play a role in calculating a staged volume limiting approach to scheduling patients’ surgeries. Fortunately, the personal protective equipment (PPE) shortage has improved since the start of the pandemic, when due to severe limitations, elective surgeries were put on hold and only emergent cases were prioritized and performed, but now there are new challenges due to magnitude of COVID-19 cases overall.

The type of pituitary disease is also an important consideration to determine need for surgery. The Pituitary Society (1) proposed stratifying cases as emergent, urgent, or elective. Pituitary apoplexy with acute severe visual loss, or other significant mass effect, or possible malignant pathology should be considered emergent or urgent. The approach in pituitary apoplexy differs from center to center and country to country; for example, initial use of high-dose dexamethasone has been suggested even in non-pandemic times in patients without severe visual loss (4, 6). Involution of a pituitary adenoma after pituitary apoplexy has also been reported in a patient whose surgery was delayed during the COVID pandemic (44).

A case-by-case basis decision for urgent surgery should be made for patients with slowly progressive visual loss, functioning tumors with aggressive clinical features, if not controlled by medical therapy. Other types of tumors, either functioning ones, well controlled on medical therapy or nonfunctioning adenomas can be scheduled as elective cases (6).

Testing for COVID-19 at 48–72 h prior to any surgery is advised (45, 46), especially for pituitary surgery where risks are higher for patients, but also for staff (43, 47). Intensified preoperative screening, even in asymptomatic patients, reverse transcription polymerase chain reaction (rRT-PCR) for all symptomatic cases, and an increased use of airborne PPE was reported in a large international survey to associated with decreased reports of COVID-19 transmission during transsphenoidal surgery (TSS) (45). Interestingly in the survey, patients were reportedly asymptomatic 32% of the time in cases of healthcare transmission (45).

A postoperative outcomes study of patients diagnosed with COVID-19 peri-operatively, found 14/51 (27.5%) postoperative mortality rate and severe mostly pulmonic complications, as well as high medical staff exposure and transmission (46). If a patient is positive for SARS-CoV-2, surgery is usually delayed at least 30–45 days unless needs are urgent or there is worsening clinical status that cannot be medically controlled. Interestingly, safe TSS has been reported in a COVID-19 positive patient presenting with pituitary apoplexy few days after inducted vaginal delivery (48).

### Cushing’s Disease

In patients with high suspicion of hypercortisolemia, testing should be performed accordingly. While initially postponing some tests in the height of the pandemic was recommended (1, 5), the longer duration of the status quo could be detrimental to patients who typically, have already experienced a long delay in diagnosis. Overall step-wise testing, screening, confirmation and localization is still suggested. However, a few tests can be performed at the same time rather than sequentially. At our Center, for patients evaluated by telemedicine with high suspicion of CS, two salivary cortisol and urinary free cortisol (UFC) are ordered. When a patient drops them at the laboratory, adrenocorticotropic hormone (ACTH) and other testing can be checked as needed. Initial concerns related to risk of COVID-19 transmission with salivary cortisol kits (5) were assuaged. Salivary cortisol remains an important screening test with proper precautions (1). Once a CS diagnosis is made, because immunosuppression and thromboembolic disorders are common CS features, cortisol-lowering medical therapy, osilodrostat, ketoconazole, metyrapone, which are usually rapid acting (within hours or days), or etomidate, when immediate cortisol control is required, should be temporarily used (5). Treatment should be individualized and selected based on patients’ clinical features, comborbidities, drug availability and drug-drug interactions (49). Patients should have disease and treatment education/teaching online, preferably by video and numerous follow-up visits are needed initially to up-titrate doses until eucortisolemia is achieved. “Block-and-replace” has been suggested as a possible better suited treatment regimen for some patients when there is a high risk of COVID-19 in the region (1, 5). With a prolonged pandemic, periods in between peaks with more access to operating rooms and beds should be actively sought out. Patients who do not tolerate or are not controlled on medical therapy should be further selected for referral planning to surgery, especially for adrenal CS, but also CD. For ectopic CS, initial recommendations suggested a possible wait of 3–6 months for low grade neuroendocrine tumors (pancreatic or lung) (5). However, these timelines have passed now and an active plan for surgery at the same time restrictions are partially lifted is advisable.

### Acromegaly

Patients with acromegaly have frequently associated comorbidities such as cardiovascular complications, DM, restrictive pulmonary disease, and obstructive sleep apnea (27, 50), all of which are associated with higher risk of complications if COVID-19 infected (see above). Furthermore, delay in diagnosis is already very long in these patients even in non-pandemic circumstances, up to 11 years in some cases. A diagnosis could be made *via* telemedicine evaluation (preferably by video) and screening/confirmatory laboratory work-up. An elevated IGF-1 in a patient with clinical features of GH excess should promptly trigger the need for imaging and treatment. Correspondingly, for any patients with newly diagnosed pituitary adenoma, hormonal laboratory work-up should include IGF-1 to rule out GH excess (6).

If imaging shows a pituitary tumor that is not compressing optic chiasm, while planning for surgery (see above) depending on hospital and region characteristics, medical therapy should be at least considered. During the pandemic, at our Center, we have treated many patients with newly diagnosed GH excess with primary therapy, either SRLs or pegvisomant. Most patients treated with SRLs had overall larger tumors and did not have severe DM; patients were started on injections at higher doses (rather than the usual slow up-titration), lanreotide 120 mg or octreotide 30 mg. While octreotide requires nurse administration, lanreotide can be safely self- or partner-administered at home in most cases. Nurse teaching for injections either in clinic or by video has been helpful for patients to increase their confidence and to supervise a first injection. A few patients with small tumors and comorbidities, as diabetes for example, could be treated initially with pegvisomant (1, 2, 6, 50, 51), which has a subcutaneous administration.

For patients on long-term medical treatment when the pandemic started, some adjustments have been also helpful, for example increasing SRLs doses, which allowed for a longer injection interval (5–6 weeks or even more) (1, 2, 6, 51) for patients who could not have injections at home. In the US, oral octreotide has been Food and Drug Administration approved and available since the Fall of 2020, thus allowing some patients controlled on injectable SRLs (but requiring nurse visits for administration) to switch to an oral therapy (52).

Close monitoring is needed for all patients, either on stable therapy or especially after changes are made; serial IGF-1 and GH (except for patients on pegvisomant), safety testing in conjunction with signs or symptoms elicited during a telemedicine visit.

Many investigations required for screening of acromegaly related complications (27, 30) have been almost completely arrested. It is essential that patients are advised and even scheduled in advance wherever possible, to undergo screening when hospital systems open up, or even in between COVID-19 low and high infection rates.

### Prolactinomas

Most patients with prolactin-secreting tumors can be medically treated with dopamine agonists (DAs) (50). However, these drugs are now recognized to increase risk of depression and impulse control disorders (ICDs) (53, 54) and mental health is understandably, more so affected during a pandemic. It is critical that both comprehensive psychiatric history and concomitant medications are acquired before starting DAs (1); specific questions might be needed to elicit response, especially if visits are undertaken virtually. Patients with larger tumors who need treatment and have severe depression at baseline would require multidisciplinary management with psychiatry and close monitoring. Serial virtual visits every 2 weeks have been helpful for clinical monitoring and dose adjustments in the authors’ personal experience.

### Adrenal Insufficiency

Counseling on sick-day rules and to start GC stress dose treatment immediately at the onset of symptoms suspicious for COVID-19 (even if not confirmed) in patients with known AI is essential. Furthermore, these doses should be continued until symptom resolution. If a COVID-19 infection is confirmed, depending on severity of symptoms doses will vary (see above). Confirming with patients at every visit their home supply of both oral and injectable GC increases compliance and reinforces the importance of adrenal crisis prevention with patients and their family (1, 2, 6, 51).

### Growth Hormone Deficiency

Relatively surprisingly, data shows that most patients with GH deficiency of all ages, including children and adults, but also transition patients had overall good adherence to GH therapy during the COVID-19 pandemic (55). The authors’ explanation of this better than expected adherence in the pandemic compared with previous data was linked to home confinement despite the disruption of clinical care for many patients.

## Conclusion

Reduced access to day-to-day regular clinical services for patients with suspected or confirmed pituitary disease, could create delays in both diagnosis and treatment plans. A modified management approach is needed for patients who require regular/routine monitoring, which likely will vary from country to country depending on health care systems and virus load.

Patients with pituitary disorders should be stratified by risk and virtual follow-ups generally recommended to further evaluate any changes. Many of these patients have hypopituitarism including secondary AI, requiring stress-dose GC. For patients with hyper-functioning tumors; acromegaly, CD, and prolactinomas, laboratory testing, though initially postponed for few months at the beginning of the pandemic is needed to ensure patients are well controlled on therapy or that the disease did not recur. Dose adjustments can be made based on clinical assessment and telemedicine virtual visits in the interim. A tight balance between minimizing frequency to reduce infection risk and monitoring for tumor growth is also required for pituitary imaging in patients with more aggressive tumors. A year into the pandemic, change in how patients are treated is still needed. To prevent an increase in complications and patient satisfaction, motivation, but also adherence, care delivery standardization for pituitary disorders is advised in this crisis. Hope is, however, on the way with multiple vaccination programs. Restoration of highly specialized work in Pituitary Centers following COVID-19 and planning for new service delivery and research should take into account all challenges encountered during the pandemic. Hopefully, in the end, we will create a novel healthcare delivery system better suited to the presumed post-COVID-19 environment.

## Author Contributions

The author confirms being the sole contributor of this work and has approved it for publication.

## Conflict of Interest

MF has received research support as principal investigator to Oregon Health & Science University from Chiasma, Crinetics, Ionis, Recordati, and Strongbridge and as an occasional scientific consultant from Chiasma, Crinetics, Ionis, Ipsen, Pfizer, Recordati, and Strongbridge.
